# Impact Exerted by Nutritional Risk Screening on Clinical Outcome of Patients with Esophageal Cancer

**DOI:** 10.1155/2018/7894084

**Published:** 2018-03-27

**Authors:** Rui Wang, Hongfei Cai, Yang Li, Caiwen Chen, Youbin Cui

**Affiliations:** Department of Thoracic Surgery, The First Hospital of Jilin University, 71 Xinmin Street, Changchun, Jilin 130021, China

## Abstract

**Objective:**

Preoperative nutritional status of patients is closely associated with their recovery after the surgery. This study aims to ascertain the impact exerted by the nutritional risk screening on clinical outcome of patients with esophageal cancer.

**Methods:**

160 patients with esophageal cancer aged over 60, having got therapy at the First Hospital of Jilin University from Jun 2016 to Feb 2017 were evaluated by adopting the NRS2002. 80 cases of patients got active therapy of nutritional support, and the other patients not supported nutritionally were selected as the control group. The comparison was drawn between two groups in serum albumin, serum immunoglobulin, postoperative complications, hospitalization, and hospitalization expenses.

**Results:**

For all the patients, in 3 and 7 days after the surgery, the serum albumin in the nutritionally supported group outstripped that in group without nutritional support (*P* < 0.05) regardless of the nutritional risk. For the patients in the risk of nutrition, the IgA in the nutritionally supported group outstripped that of group without nutritional support (*P* < 0.05) in 3 and 7 days before the surgery, and the serum IgG outstripped that of the group without nutritional support in 1 and 3 days before the surgery (*P* < 0.05). In terms of the patients in the risk of nutrition, the average hospitalization of nutritionally supported group was shorter (*P* < 0.05), and the average hospitalization expenses were lower compared with those of the group without nutritional support. And for the patients in no risk, the hospitalization expenses of supported group surmounted those of group without nutritional support (*P* < 0.05), whereas the average hospitalization took on no statistic difference (*P* > 0.05).

**Conclusion:**

For the patients in the risk of nutrition, preoperative nutritional support can facilitate the nutritional status and immunization-relative result after surgery, which shall also decrease the average hospitalization and hospitalization cost.

## 1. Introduction

Esophageal cancer refers to the sixth malignant tumor most commonly occurring worldwide, the surgical treatment-based therapy counts as the primary treatment for esophageal cancer [1–3]. The therapeutic effect on esophageal cancer has been evidently improved; yet the prognosis remains difficult to acquire satisfactory results. Esophagectomy counts as a type of invasive surgery, and obstructions of the food passageway and the overgrowth of tissue postoperatively may cause malnutrition [4]. As reported in the previous research, malnutrition develops in 79% patients with esophageal cancer [5]. Malnutrition can make patients react with postoperative resistance stressfully. It shall raise the rate of complication and mortality, and malnutrition also counts as an independent risk factor for the hospitalization and hospital costs. In this regard, nutrition evaluation and management for patients with esophageal cancer is of critical significance for the recovery of patients after surgery [6].

NR2002 (Nutritional Risk Screening 2002) has been used to measure the nutritional status of patients (stroke patients in North China) in previous studies which has a good reliability [7]. In this study, NRS2002 was adopted to screen the aged patients with esophageal cancer over the age of 60, and the corresponding nutritional branches were established following the screening results. The aim was to assess the impact of nutritional support on clinical outcomes in elderly patients with esophageal cancer.

## 2. Methods

### 2.1. Subjects

This study recruited patients aged over 60 in Bethune First Hospital affiliated by Jilin University from Jun 2016 to Feb 2017. After signing the consent, 160 patients were included in this study according to the inclusion and exclusion criteria.

### 2.2. Inclusion Criteria

1. Patients aged over 60
2. Patients diagnosed with malignant esophageal tumor and with indications of surgery (without cancer metastasis)

### 2.3. Exclusion Criteria

1. Patients with esophageal cancer taking on metastasis from other sites
2. Patients who were unwell to be engaged in this program

### 2.4. Nutritional Risk Screening

The nutritional risk was screened for all patients in this study through adopting NRS2002 [8]. 80 cases of patients got therapy of nutritional support, and the other patients without nutritional support were selected as the control group. Patients scored over 3 are defined as in the risk of nutrition. As the screening result indicates, 104 cases are in the risk of nutrition.

### 2.5. Nutritional Support Program

Patients have a routine diet following the advice of doctor before the surgery: milk, broth, soy milk, and other liquid-based, nonslag, and high nutrient solution. Patients in the nutritionally supported group were given nutritional intervention by routine nursing before the surgery. The nutrient solution supplemented appropriate electrolyte and other trace elements and is supplied according to heat calculation method and proportion 20 kCal/kg per day 3 days before surgery; thermal nitrogen ratio was 150 kCal : 1 g; the sugar-lipid mass ratio was 1 : 1. During hospitalization, the clinicians collect the nutritional intake of all the patients on time.

Postoperative nutritionally supported group and group without nutritional support were provided with energy (25 Kcal/kg per day) through the duodenal nutrient tube (intraoperative implantation). This study was approved by the Ethics Committee of the First Hospital of Jilin University, and written informed consent was obtained from all the subjects in the study.

### 2.6. Statistical Analysis

The serum albumin and serum immunoglobulin of patients were collected 1 day before the surgery and 1, 3, and 7 days after the surgery. Furthermore, the situation of complications, hospitalization, and expense were also collected. All the data were anatomized adopting IBM SPSS 19.0 (ver. 19.0; IBM Corp, Armonk, NY, USA), *t*-test and *χ*^2^ test were adopted to compare the difference between groups, and *P* < 0.05 was considered to be statistically significant.

## 3. Result

160 patients esophageal cancer aged over 60 with indication of surgery were involved in this study. As indicated in Table 1, 144 males and 16 females were the entire patients, among which 144 were subject to squamous cell carcinoma, 12 were subject to adenocarcinoma, 3 cases suffered from squamous cell carcinoma, and 1 suffered from small cell carcinoma. The nutritional risk screening was screened for all the patients in this study through adopting NRS2002. 104 patients were in the risk of nutrition, and the detailed information was presented below.

The results of comparing serum albumin and serum immunoglobulin in patients in the risk of nutrition are listed in Table 2. Serum albumin and serum IgA in the nutritionally supported group were evidently more than those in the group without nutrition support 3 and 7 days after the surgery. Serum IgG in nutritionally supported group was significantly better than the group without nutrition support 1 and 3 days after surgery.

The results of the comparison of serum albumin and serum immunoglobulin in patients not in the risk of nutrition are listed in Tables 3 and 4. Serum albumin and serum immunoglobulin (IgA and IgG) were both significantly better than the group without nutrition support 3 and 7 days after the surgery.

We finally compared the situation of complication, hospitalization in the hospital, and medical expense. Among patients who have the nutritional risk, the length of nutritionally supported group was shorter than the group without nutrition support, whereas the medical expenses are more, so the situation exists among the patients in the risk of nutrition. The detailed information is in Table 5.

## 4. Discussion

Esophageal cancer is deemed as one of the most commonly occurring malignant tumors in China; the incidence of this disease has increased considerably in the past few years [9, 10]. Surgery counts as the primary treatment strategy presently. Surgical techniques have leaped forward after decades of development. The total resection rate and the 5-year survival rate were evidently improved [11, 12]. Yet in Chinese patients with esophageal cancer, especially in elderly patients, most of the patients face difficulties in eating during the middle and late stages. Arising from the malnutrition caused by eating difficulties and tumor consumption, most patients took on poor nutritional status before the surgery [13]. As reported by previous research, malnutrition counts as an independent risk factor affecting the prognosis of surgery, which shall evidently raise the complication and morbidity after surgery. It can also cause a bulk of adverse effects, inclusive of prolonged hospitalization, increased cost, slower recovery, and lower quality of life [6, 14, 15] In this regard, evaluating the nutritional status of patients with esophageal cancer is of crucial significance, especially those who are ready to get surgery, and we seek to formulate a judicious nutritional support plan.

NRS2002 is a nutritional risk screening tool recommended by ESPEN. The data were derived from 128 randomized controlled trials which combined BMI, treatment and malnutrition, recent body mass changes, and recent changes in nutritional intake. This assessment has been validated by several research institutes and has been recommended as the preferred tool for nutritional risk assessment in hospitalized patients. ESPEN recommends nutritional support for patients in the risk of nutrition [16]. In this study, patients in the nutritional support group received nutritional support 3 days before the surgery.

Although the levels of albumin and immunoglobulin in patients in the risk of nutrition 1 day before surgery were lower than those without nutritional risk, postoperative data indicates that patients who received nutritional support recovered faster than those without nutritional support. Additionally, given patients nutritional support 3 days before surgery did not improve the overall cost of hospitalization. In the two groups of patients in the risk of nutrition, the cost of hospitalization decreased. It is directly related to the nutritional status of patients after surgery that good nutritional status stimulated patients' recovery and decreased average hospitalization time. Therefore, we believe that, for patients in the risk of nutrition, preoperative nutritional support therapy does not bring greater economic burden to the patients.

For patients without nutritional risk, serum albumin and immunoglobulin for patients with nutritional support were found to outstrip those who are without nutritional support. Yet the incidence of complications and hospitalization in the nutritionally supported group were not evidently advantaged, and the cost was higher. Therefore, providing nutritional support for patients without nutritional risk before surgery should comply with the specific condition of patients.

As this study indicated, the preoperative nutritional support for patients takes on evident therapeutic effect on facilitating their nutritional status. As some studies assert to facilitate the postoperative state and reduce the incidence of postoperative complications and mortality of patients subject to serious malnutrition, nutritional support therapy is required to be given in excess 10 days before the surgery [17]. Yet some researches uphold that nutritional support over 7 days before the operation may stimulate tumor growth and proliferation, and an underlying risk of increasing tumor metastasis is posed. In this regard, they did not recommend supporting nutrition for long periods [18, 19]. In this study, nutritional support therapy was performed 3 days before surgery; the main consideration is that prolonged nutritional support increases tumor growth rates and longtime preparation also increases the risk of tumor metastasis.

Previous studies have shown that many of the patients with esophageal cancer are from low-income families [20]. The cost of hospitalization and surgery brings a great financial burden to their families. This study found that improving the nutritional situation and immunization-relative result after surgery can decrease the average hospitalization and hospitalization cost. Therefore, this study can provide a complementary opinion to reduce the financial burden of the patients. The result shows the effectiveness of nutritional support program, but how long before surgery should the nutritional support be given has not been analyzed, so further study is still needed.

## 5. Conclusion

For the patients in the risk of nutrition, nutritional support before surgery can improve the nutritional situation and immunization-relative result after surgery, which can also decrease the average hospitalization and hospitalization cost.

## Figures and Tables

**Table 1:** Characteristics of patients included in this study.

Character	*N*	%
Age		
60–70	112	70.0
>70	48	30.0
Sex		
Male	144	90.0
Female	16	10.0
Pathological type		
Squamous cell carcinoma	144	90.0
Adenocarcinoma	12	7.5
Adenosquamous carcinoma	3	1.9
Small-cell carcinoma	1	0.6

**Table 2:** Comparison of serum immunoglobulin and serum albumin between two groups of patients with nutritional risk.

Items	Nutrition support (*n* = 52)	Nonnutrition support (*n* = 52)	*t*	*P*
IgA				
1 day before operation	2.21 ± 0.37	2.18 ± 0.56	0.28	0.780
1 day after operation	2.01 ± 0.17	1.98 ± 0.21	0.80	0.425
3 days after operation	2.15 ± 0.33	2.03 ± 0.24	2.12	0.036
7 days after operation	2.19 ± 0.14	2.09 ± 0.11	4.05	<0.01
IgG				
1 day before operation	10.14 ± 0.52	10.35 ± 0.94	−1.41	0.162
1 day after operation	8.77 ± 0.77	8.32 ± 1.01	2.83	0.006
3 days after operation	8.41 ± 0.41	8.09 ± 0.85	2.45	0.016
7 days after operation	8. 37 ± 0.16	8.3 ± 0.33	1.38	0.172
Serum albumin				
1 day before operation	35.4 ± 2.8	35.8 ± 3.2	−0.68	0.499
1 day after operation	31.3 ± 1.7	30.9 ± 2.0	1.10	0.274
3 days after operation	33.6 ± 2.1	32.1 ± 3.2	2.83	0.006
7 days after operation	35.1 ± 1.7	33.9 ± 2.4	2.94	0.004

**Table 3:** Comparison of serum albumin between two groups of patients who have no nutritional risk.

Group	*N*	Serum albumin			
		1 day before operation	1 day after operation	3 days after operation	7 days after operation
Nutrition support	52	38.2 ± 2.6	33.1 ± 1.9	36.6 ± 2.3	38.1 ± 2.7
Nonnutrition support	52	37.9 ± 2.1	32.9 ± 2.0	34.1 ± 3.4	36.2 ± 2.1
*t*	-	0.65	0.52	4.39	4.01
*P*	-	0.519	0.602	<0.001	<0.001

**Table 4:** Comparison of Serum immunoglobulin between two groups of patients who have no nutritional risk.

Items	Nutrition support	Nonnutrition support	*t*	*P*
IgA				
1 day before operation	2.41 ± 0.31	2.35 ± 0.76	0.37	0.711
1 day after operation	2.12 ± 0.42	2.07 ± 0.37	0.46	0.651
3 days after operation	2.09 ± 0.53	1.92 ± 0.44	1.26	0.214
7 days after operation	2.23 ± 0.66	1.93 ± 0.31	2.10	0.041
IgG				
1 day before operation	12.34 ± 0.45	12.77 ± 0.73	2.56	0.014
1 day after operation	10.43 ± 0.54	10.67 ± 0.69	1.40	0.169
3 days after operation	9.21 ± 0.44	8.33 ± 0.62	5.90	<0.001
7 days after operation	9.32 ± 0.19	8.45 ± 0.58	5.32	<0.001

**Table 5:** Comparison of complications and hospitalization information between two groups of patients with/without nutritional risk.

Group	*n*	Complications	Length of stay	Medical expenses
Patients with nutritional risk				
Nutrition support	52	8 (15.38)	12.4 ± 3.9	93723 ± 5614
Nonnutrition support	52	9 (17.30)	14.2 ± 3.1	95988 ± 4612
*χ*^2^/*t*		0.07	2.61	2.25
*P*		0.791	0.011	0.027
Patients without nutritional risk				
Nutrition support	28	4 (14.28)	11.6 ± 3.4	92628 ± 5013
Nonnutrition support	28	5 (17.85)	12.1 ± 3.1	89748 ± 4779
*χ*^2^/*t*		0.13	0.78	3.00
*P*		0.716	0.435	0.003
